# Schwann cell hamartoma: case report

**DOI:** 10.1186/1471-230X-11-68

**Published:** 2011-06-10

**Authors:** Elena Guerini Rocco, Francesca Iannuzzi, Alessandra Dell'Era, Monica Falleni, Laura Moneghini, Franca Di Nuovo, Paola Braidotti, Gaetano Bulfamante, Solange Romagnoli

**Affiliations:** 1 Department of Medicine, Surgery and Dentistry, Division of Pathology, A.O. San Paolo and Fondazione IRCCS Cà-Granda Ospedale Maggiore Policlinico, University of Milan Medical School, Milan, Italy; 2 Department of Clinical Sciences "Luigi Sacco", University of Milan Medical School, Milan, Italy; 3 Azienda Ospedaliera G.Salvini, Garbagnate Milanese, Italy

**Keywords:** Schwann cell, hamartoma, schwannoma, gastrointestinal stromal tumors, colonic polyp

## Abstract

**Background:**

Colorectal polyps of mesenchymal origin represent a small percentage of gastrointestinal (GI) lesions. Nevertheless, they are encountered with increasing frequency since the widespread adoption of colonoscopy screening.

**Case presentation:**

We report a case of a small colonic polyp that presented as intramucosal diffuse spindle cell proliferation with a benign cytological appearance, strong and diffuse immunoreactivity for S-100 protein, and pure Schwann cell phenotype. Careful morphological, immunohistochemical and clinical evaluation emphasize the differences from other stromal colonic lesions and distinguish it from schwannoma, a circumscribed benign nerve sheath tumor that rarely arises in the GI tract.

**Conclusion:**

As recently proposed, this lesion was finally described as mucosal Schwann cell hamartoma.

## Background

Polypoid mesenchymal lesions of the colon are increasingly being detected because of growth in the number of colonoscopic procedures for colorectal cancer screening [1]. The histological differential diagnosis of these lesions is broad and includes gastrointestinal stromal tumors (GISTs), fibrous lesions, and neoplasms of smooth muscle and neural origin [2].

Neural lesions of the colon are a rare group of colorectal disorders that may frequently present as small polyps [3]. This heterogeneous group includes ganglion cells and nerve sheath tumors that have been frequently described as multiple lesions in association with inherited syndrome such as von Recklinghausen's (or type 1) neurofibromatosis (NF-1) [4,5], multiple endocrine neoplasia type 2B (MEN 2B) [6] and Cowden syndrome [7]. Moreover, sporadic forms of these neural lesions have been reported including solitary colonic polypoid ganglioneuroma [8], solitary colonic neurofibroma [9,10], and mucosal benign epithelioid nerve sheath tumors [11].

Recently, Gibson and Hornick have analysed 26 cases of colorectal polypoid lesions comprising pure Schwann cell proliferation, which were different from gastrointestinal tract (GI) schwannomas, and with no association with inherited syndromes. This entity was designated as mucosal Schwann cell hamartoma [3].

In this report, we describe a case of a polypoid lesion of the colon with features of this recently proposed new entity.

## Case presentation

A 67-year-old woman underwent routine follow-up colonoscopy after resection of colonic tubulovillous adenoma. The patient had a colonoscopy with biopsy at the same institution 1 year previously. At that time, a bulky lesion (4.5 cm wide) of the descending colon was identified and diagnosed histologically as tubulovillous adenoma with low-grade dysplasia. As a result of the large dimension of the adenoma, the patient underwent resection of the transverse-descending colon, which confirmed the diagnosis of tubulovillous adenoma with low- and high-grade dysplasia.

Follow-up colonoscopy was performed to reach the cecum; the anastomosis was pervious and covered by normal appearing mucosa. At 30 cm from the anal rim one small sessile polyp, 3 mm in size was removed by cold biopsy. The other parts of the intestine showed normal appearing mucosa.

The patient had no family or personal history of signs or symptoms related to other neural lesions or inherited syndromes such as NF-1, Cowden syndrome or MEN 2B.

**Figure 1:**
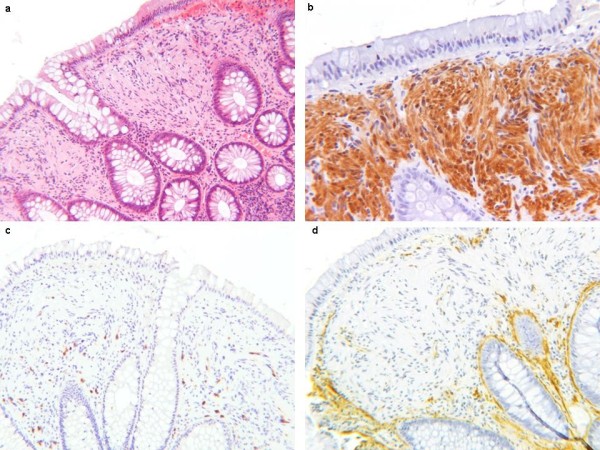
**Histological features of the lesion showing colonic mucosa with diffuse Schwann cell proliferation within the lamina propria**. Spindle cells with elongated nuclei and no cytological atypia; low-power magnification, H&E staining (a); the lesion shows strong immunoreactivity for S-100 protein (b) and no immunoreactivity for CD117 (c) or smooth muscle actin (d).

Histological analysis of hematoxylin and eosin (H&E)-stained sections of the sample showed a diffuse proliferation of spindle cells in the colonic mucosa, located in the lamina propria that separates and distorts the colonic crypt architecture. This lesion was poorly circumscribed and the muscularis mucosa was not involved (Figure 1a). The surface epithelium was intact without ulceration or erosion. The lesion had a homogeneous and benign cytological appearance. All cells were spindle shaped with elongated, tapering nuclei, abundant dense eosinophilic cytoplasm with indistinct cell borders, and with no nuclear atypia, pleomorphism or mitoses.

At immunohistochemistry all cells displayed strong positivity for S-100 protein (Figure 1b) and no immunoreactivity for CD34 (QB END/10), CD117 (KIT) (Figure 1c), or smooth muscle actin (Figure 1d). The patient is due for computed tomography and colonoscopy at 1 year.

## Conclusions

Since colonoscopy was recognized as the gold standard screening procedure for colorectal cancer, the increasing number of GI biopsy or polypectomy samples has led to pathologists frequently encountering lesions that are different from epithelial neoplasms. As a result of the large spectrum of histological types and their different clinical, therapeutic and prognostic implications, accurate recognition and differential diagnosis are crucial. In this report, we describe a polypoid mesenchymal lesion of the colon consisting of poorly circumscribed proliferation of spindle cells in the lamina propria, with benign cytological appearance and by pure Schwann cell immunophenotype (S100^+^, CD34^-^, CD117^-^, smooth muscle actin and desmin^-^).

Endoscopically, the lesion showed an indefinite pit pattern and the mucosal crypts on the surface of the lesion could not be evaluated during colonoscopy. According to the modified mucosal crypt pattern criteria, it is possible to distinguish five types: type I, round pit; type II, stellar or papillary pits; type III, tubular or small roundish pits; type IV branch-like or gyrus-like pits; and type V, non-structural pits. Types I and II distinguish non-neoplastic lesions from type III, IV or V crypt patterns that are considered neoplastic [12,13]. These criteria can predict histopathology of the lesions by magnifying or non-magnifying colonoscopy [13].

Stellar, asteroid pits distinguish hyperplastic polyps, while branching pits are typical of adenomas [12]. In the case of non-epithelial lesions, such as in Schwann cell hamartoma, the pit pattern should be of non-neoplastic type. Moreover, the evaluation of the vasculature pattern of colonic lesions highlighted by magnifying narrow band imaging may help in distinguishing a mesenchymal lesion from other more common colorectal lesions, for which the method is applied [14].

The histological differential diagnosis of spindle cell proliferation that may present as colon polyp is broad and includes GISTs, fibrous lesions, and neoplasms of smooth muscle [2]. GISTs are more commonly found in the stomach and small intestine but a small percentage occurs in the colon, and their recognition is important due to the variable malignant clinical course and the response to targeted therapy. GIST may display neural features with S100 protein expression but they are characterized by peculiar immunoreactivity to C-KIT/CD117 antibodies [2,15]. Inflammatory fibroid polyps (Vanek's tumor) [16] and benign fibroblastic polyps [17] are benign fibrous lesions in which proliferating spindle cells typically display perivascular arrangement, diffuse immunoreactivity for vimentin, and characteristic inflammatory infiltrates.

Recently the entities of fibroblastic polyp/colonic perineuroma have been recognized as the same lesion; they display bland, spindle-shaped cells separating the mucosal crypt, and show serrated crypt architecture on top of or adjacent to the lesion. The predilection site is the rectosigmoid colon and the lesion is more frequently encountered in women. The hypothesis has been confirmed by the immunohistochemical evaluation of fibroblastic polyps/perineuromas with markers of perineural differentiation, that, is claudin-1, GLUT-1, collagen type IV and epithelial membrane antigen (EMA), and partially confirmed by electron microscopy [18]. In a significant number of cases (63%), a V600E BRAF mutation is present, and less frequently, a KRAS mutation [19]. The proposed pathogenesis of serrated fibroblastic polyps/intramucosal perineuromas is the perineural stromal component that arises from epithelial-stromal interaction with proliferation of pericryptic fibroblasts. Different from perineuroma, our case showed a strong immunoreactivity for protein S-100 and lacked the serrated glands.

Leiomyoma can also arise in association with muscularis mucosae and present as a polypoid lesion but display a smooth muscle cell immunophenotype that is strongly and diffusely positive for desmin and actin [20]. The homogeneous and intense immunoreactivity to S100 antibody in our case and negativity for actin, desmin, CD34 and CD117 clarified the neural origin of the lesion. In 2009, Gibson and Hornick reported a series of 26 neural colorectal polyps similar to our case and distinct from other nerve sheath tumors [3].

The lack of axons and ganglion cells argues against lesions such as neurofibroma, ganglioneuroma or mucosal neuroma [21]. Moreover, these tumors display significant association with inherited syndromes. Colorectal neurofibroma is nearly pathognomonic of NF1, mucosal neuroma is highly associated with MEN 2B, and ganglioneuroma occurs frequently in association with Cowden syndrome, MEN 2B or NF1 [4-7]. However, none of these clinical features was found in the present case. Also intramucosal perineuroma can display morphological features very similar to our case, but lack of S100 expression and immunoreactivity to EMA aids the proper differentiation of these lesions [22].

Finally, the diagnosis of intramucosal schwannoma has to be considered. Schwannomas represent a small percentage of GI mesenchymal tumors and rarely occur in the colon. In contrast to their soft tissue counterpart, nuclear palisading, typical Verocay bodies, vascular hyalinization and xanthoma cells are not seen [23,24]. Colon schwannoma could present as polypoid intraluminal mass but intramural extension, more circumscribed margins, and characteristic peripheral lymphoid cuffs differentiate it from the lesion described [23,24]. According to the new entity proposed by Gibson and Hornick [3], the case reported here was finally diagnosed as mucosal Schwann cell hamartoma. Accurate histological analysis and an appropriate immunohistochemical panel are sufficient to identify this lesion and to distinguish it from other colon polypoid lesions of mesenchymal origin.

## Competing interests

The authors declare that they have no competing interests.

## Authors' contributions

All authors read and approved this manuscript. EGR analyzed and interpreted the patient data, and drafted the manuscript; FI performed the endoscopy; ADE followed up the patient; LM, MF and FDN revised the pathological data; PB performed the immunohistochemical analysis; and GB and SR supervised the case report.

## Consent

Written informed consent was obtained from the patient for publication of this case report and any accompanying images. A copy of the written consent is available for review from the Editor-in-Chief of this journal.

## Pre-publication history

The pre-publication history for this paper can be accessed here:

http://www.biomedcentral.com/1471-230X/11/68/prepub
